# Bochdalek hernia presenting as recurrent fever and bicytopenia in a pediatric patient: A rare case report

**DOI:** 10.1002/ccr3.8039

**Published:** 2023-10-10

**Authors:** Pouria Salajegheh, Abolfazl Gilani, Farzaneh Yazdi, Roham Sarmadian, Adrina Habibzadeh

**Affiliations:** ^1^ Department of Pediatric Hematology & Oncology Kerman University of Medical Sciences Kerman Iran; ^2^ Sina Trauma & Surgery Research Center Tehran University of Medical Sciences Tehran Iran; ^3^ Endocrinology and Metabolism Research Center Kerman University of Medical Sciences Kerman Iran; ^4^ Infectious Diseases Research Center Arak University of Medical Sciences Arak Iran; ^5^ Student Research Committee Fasa University of Medical Sciences Fasa Iran; ^6^ USERN Office Fasa University of Medical Sciences Fasa Iran

**Keywords:** Bochdalek hernia, diaphragmatic hernia, emergency care, thrombocytopenia

## Abstract

Bochdalek hernia is a rare condition characterized by the displacement of abdominal contents into the thoracic cavity. Due to the nonspecific nature of symptoms, prompt diagnosis, and management by emergency care providers can be challenging. Treatment of a Bochdalek hernia typically involves the reduction in the herniated contents back into the abdominal cavity. In this case report, we present the case of a 1‐year‐old girl who presented to the emergency department with a fever and bicytopenia. Further evaluation revealed a Bochdalek hernia, which was successfully managed with surgical intervention. This case highlights the importance of considering a Bochdalek hernia in the differential diagnosis of patients presenting with recurrent nonspecific symptoms (fever and bicytopenia).

## Graphical abstract

Chest X‐ray capturing the distinctive features of a Bochdalek hernia in a pediatric patient. The radiograph vividly showcases the diaphragmatic herniation and consequential shift in mediastinal structures. Bochdalek hernia, a rare condition, involves the displacement of abdominal contents into the thoracic cavity. This case report emphasizes the challenges in diagnosing and managing Bochdalek hernia, particularly in the context of nonspecific symptoms.

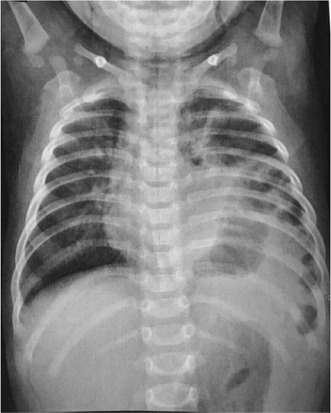

## 1 INTRODUCTION

Medical professionals responding to emergencies often encounter patients with nonspecific symptoms such as fever, which can pose a diagnostic challenge and require immediate identification of the underlying pathology. Rare or atypical conditions can pose a significant challenge for emergency department physicians, who may not often encounter them frequently in their practice. Accurate diagnosis and case‐specific treatment are essential to ensuring the best possible outcome for patients with these conditions. In 1848, Czech anatomist Vincent Alexander Bochdalek described the Bochdalek hernia.^1^ It is caused by failure of fusion between the 10th and 11th ribs on the posterolateral diaphragmatic foramen, also known as the Bochdalek foramen. This aperture connects the peritoneal and pleural cavities, so this occurs more often in newborns and young children than in adults.^2^ Herein, we presented a case of a young female who presented with nonspecific symptoms to the emergency department and was diagnosed with a Bochdalek hernia. She had her herniated spleen and a Bochdalek hernia repaired.

## 2 CASE REPORT

We report the case of a 1‐year‐old girl who presented to the emergency department with a history of fever for 3 days. The patient had no significant past medical history but had experienced flu‐like symptoms 3 weeks prior to admission. There were no symptoms of shortness of breath, chills, postnasal discharge, or abdominal pain. Vital signs were normal. Asymmetry of the chest was discovered during a physical examination. The heart sounds were normal. The slight sound decrease was detected on the left side of the lungs. The splenomegaly was revealed during an abdominal examination. And the extremities were normal. Laboratory data were obtained: White blood count: 3800/μL lymphocytes: 68% polymorphonuclear: 25%, platelet: 87000/μL, mean corpuscular volume (MCV): 76, mean corpuscular hemoglobin (MCH): 25.

The arterial blood gas (ABG) analysis revealed the following parameters: a pH of 7.38, a PaCO_2_ of 42 mmHg, an HCO_3_‐level of 22 mEq/L, a base excess of −1 mEq/L, a PaO_2_ of 75 mmHg, and a SaO_2_ of 97%.

**FIGURE 1:**
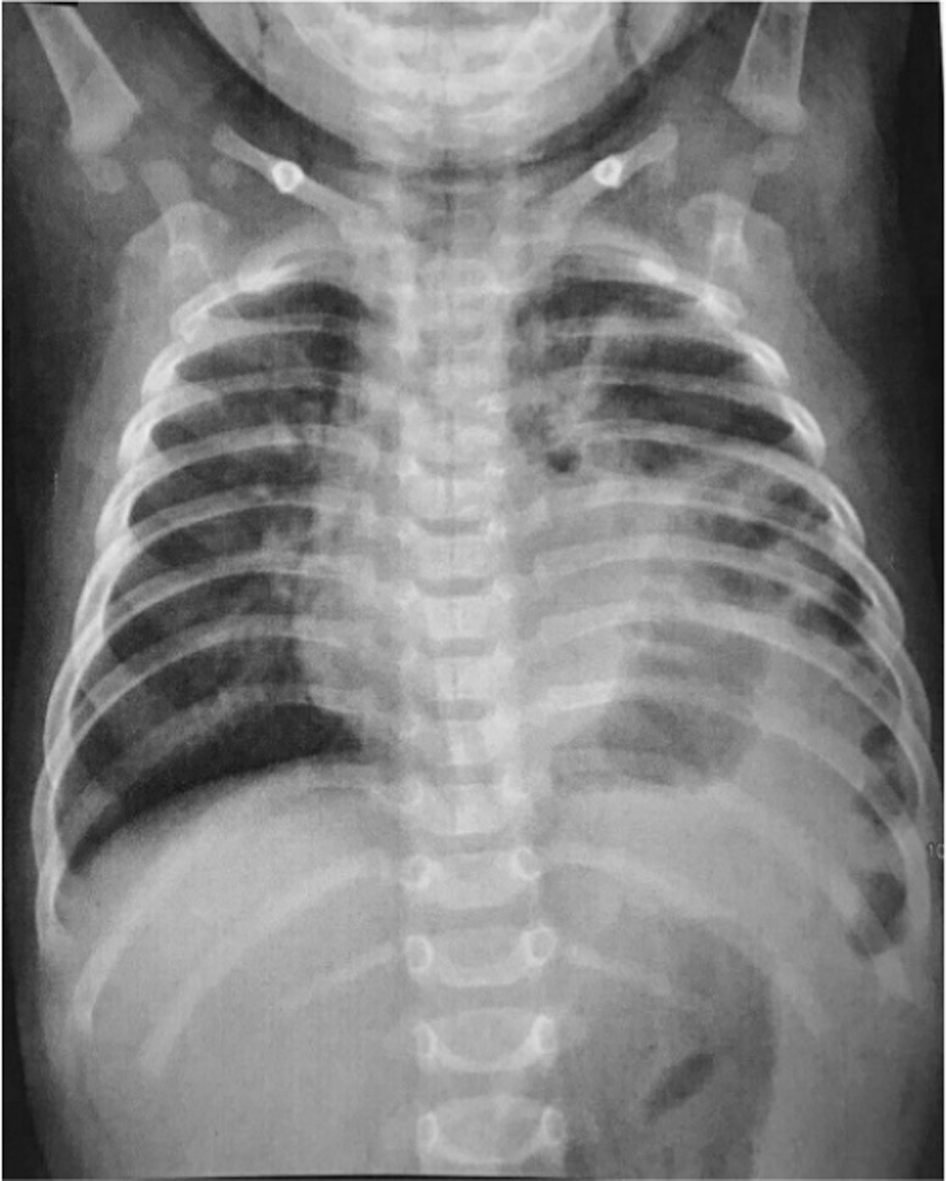
Chest X‐ray showing a diaphragmatic hernia with a shift in mediastinal structures.

Cytogenetic studies were normal with 46, XX. Due to bicytopenia and a history of recent fever, the patient consulted an oncologist, and a bone marrow biopsy was done. There was only an increase in megakaryocytes without any metastatic or immature cells. In chest X‐ray (CXR), the left side of the chest showed a diaphragmatic hernia. A shift in mediastinal structures was observed to the right (Figure 1). In meantime, due to splenomegaly and thrombocytopenia, the pediatrician suspected splenic vein thrombosis. The ultrasound Doppler imaging revealed a decrease in blood flow in the splenic vein due to thrombosis. In addition, the size of the spleen was assessed to be 10 cm by ultrasound examination. Therefore, the findings were suggestive of a Bochdalek hernia along with splenic vein thrombosis. After receiving vaccinations for pneumococcal disease and hemophilus, the patient underwent diaphragm repair and splenectomy after 8 weeks. The first part of the surgery involved addressing the Bochdalek hernia. A surgical incision was made in the appropriate location, providing access to the diaphragm. The herniated abdominal contents, which included the stomach and small intestine were carefully reduced and returned to their normal position within the abdominal cavity. The diaphragmatic defect was then repaired. Following the diaphragm repair, attention was turned toward the splenic vein thrombosis. An additional incision was made to access the spleen and the splenic vein. The surgeon carefully dissected and isolated the splenic vein. The thrombosed portion of the splenic vein was identified and carefully removed.

Once the repairs to the diaphragm and splenic vein were completed, the surgeon proceeded with the splenectomy. The spleen was dissected and removed from its attachments while ensuring minimal bleeding. The splenic artery and other associated blood vessels were carefully ligated or cauterized to prevent excessive bleeding. Once satisfied with the surgical outcome, the incisions were closed using absorbable sutures.

Postoperative recovery was uneventful, and the patient was discharged in good condition. In follow‐up, the patient's platelet count reached 700,000/μL. Complete blood count levels returned to normal within 3 months of surgery.

## 3 DISCUSSION

Sometimes, the pleuroperitoneal folds fail to seal during embryonic life, resulting in the Bochdalek aperture opening. Through this aperture, the abdominal contents herniate into the thoracic cavity, also known as the Bochdalek hernia. The defect is most evident in the first few weeks of infancy. The four forms of congenital diaphragmatic hernias are the parasternal hernia of Morgagni‐Larrey, the peritoneal–pericardial hernia, the diaphragmatic eventration, and the posterolateral hernia of Bochdalek, which is the most prevalent. The hernia is on the left side in 85% of instances. The spleen, stomach, and small and large intestines may herniate through this hernia, as seen in this case with the stomach and spleen. The majority of people are asymptomatic until they reach adulthood. Children are most likely to suffer from respiratory distress.^3^ Patients may experience chronic symptoms such as recurrent lower respiratory tract infections and chest pain. In the patient described in this case report, the presenting symptoms included flu‐like symptoms, fever episodes, and thrombocytopenia. Complications of Bochdalek hernia may include vascular compromise of herniated contents, pneumothorax, or hemothorax,^4^ but fortunately, our patient did not experience these complications.

In 25% of cases, this is completely accidental and detected on a regular chest X‐ray. However, an X‐ray can easily be misinterpreted as pulmonary sequestration, pulmonary lobar collapse, or consolidation. Because of the extensive differential diagnosis that can be made from a chest X‐ray image in two dimensions.^5^ This enables a better understanding of diaphragmatic abnormalities and their characterization.

Thrombocytopenia can be divided into two categories: increased destruction or decreased production with bone marrow hypoproliferation. Among young female patients, primary immunological thrombocytopenia, infection‐induced bone marrow suppression, drug side effects, and pregnancy thrombocytopenia are frequent causes of thrombocytopenia.^6^ Anatomical causes of thrombocytopenia exist but are rarely considered. The diaphragm generally prevents cephalad migration from the spleen. The spleen is typically locked in place in the left upper abdominal quadrant by the splenic pedicle. This pedicle is made up of the gastrosplenic and splenorenal ligaments. It houses the splenic artery and vein as well as the pancreas tail.^7^ The splenic pedicle's torsion or compression, along with the obstruction of venous outflow, is the postulated mechanism for splenic enlargement in this case. Splenic vein thrombosis is a well‐known cause of gastrointestinal bleeding and splenomegaly; the most common underlying causes are pancreatitis and pancreatic tumors.^8^, ^9^ The occurrence of isolated splenic vein thrombosis can result in a distinct clinical manifestation characterized by bleeding from isolated gastric varices, which pose challenges in terms of diagnosis, along with splenomegaly and preserved liver function.^10^ A rare consequence of congenital diaphragmatic hernia is hypersplenism from splenic sequestration leading to thrombocytopenia. Mirkes et al.^11^ describe a 21‐year‐old pregnant woman with this type of hernia. She was diagnosed with pyelonephritis and thrombocytopenia. Further evaluation revealed herniation of abdominal contents into the left hemithorax. Another study reported chronic abdominal discomfort, dyspepsia, and fatigue. Anemia and thrombocytopenia were present. CT scan of her abdomen diagnosed Bochdalek hernia, and strangulation of the spleen in the left hemithorax. This resulted in left‐sided portal hypertension, hypersplenism, and thrombocytopenia.^12^

## 4 CONCLUSION

The case presented in this report demonstrates the typical diagnosis and management of Bochdalek's hernia in an emergency setting. A combination of nonspecific symptoms and abnormal laboratory data can challenge emergency medicine practitioners. In dealing with these patients, high levels of suspicion, clinical expertise, along with a comprehensive radiological test like a CXR and Doppler ultrasonography, contribute to a definitive diagnosis and case‐specific treatment. This case highlights the importance of considering Bochdalek hernia in the differential diagnosis of pediatric patients presenting with fever and bicytopenia. Early recognition and prompt management are crucial to prevent potentially life‐threatening complications.

## AUTHOR CONTRIBUTIONS

**Pouria Salajegheh:** Conceptualization; data curation; investigation; methodology; project administration; supervision. **Abolfazl Gilani:** Validation; visualization; writing – review and editing. **Farzaneh Yazdi:** Investigation; writing – review and editing. **Roham Sarmadian:** Supervision; writing – review and editing. **Adrina Habibzadeh:** Methodology; writing – original draft; writing – review and editing.

## FUNDING INFORMATION

There is no funding to the present study.

## CONFLICT OF INTEREST STATEMENT

The authors declare that they have no conflict of interest.

## ETHICS STATEMENT

Our institution does not require ethical approval for reporting individual cases or case series.

## CONSENT

Written informed consent was obtained from the patient to publish this report in accordance with the journal's patient consent policy.

## Data Availability

All data underlying the results are available as part of the article, and no additional source data are required.
